# Development and validation of a sample entropy-based method to identify complex patient-ventilator interactions during mechanical ventilation

**DOI:** 10.1038/s41598-020-70814-4

**Published:** 2020-08-17

**Authors:** Leonardo Sarlabous, José Aquino-Esperanza, Rudys Magrans, Candelaria de Haro, Josefina López-Aguilar, Carles Subirà, Montserrat Batlle, Montserrat Rué, Gemma Gomà, Ana Ochagavia, Rafael Fernández, Lluís Blanch

**Affiliations:** 1 Critical Care Center, Hospital Universitari Parc Taulí, Institut d’Investigació i Innovació Parc Taulí I3PT, Universitat Autònoma de Barcelona, Parc Taulí 1, 08208 Sabadell, Barcelona Spain; 2 Biomedical Research Networking Center in Respiratory Disease (CIBERES), Instituto de Salud Carlos III, Madrid, Spain; 3 Facultat de Medicina, Universitat de Barcelona, Barcelona, Spain; 4 Biomedical Research Networking Center in Bioengineering, Biomaterials and Nanomedicine (CIBER-BBN), Instituto de Salud Carlos III, Madrid, Spain; 5 BetterCare S.L, Sabadell, Spain; 6 Department of Intensive Care, Fundació Althaia, Universitat Internacional de Catalunya , Manresa, Spain; 7 Department of Basic Medical Sciences, Universitat de Lleida-IRBLLEIDA, Lleida, Spain

**Keywords:** Biomarkers, Translational research, Biomarkers, Engineering, Biomedical engineering, Scientific data, Statistics, Data acquisition, Data processing, Databases, Machine learning

## Abstract

Patient-ventilator asynchronies can be detected by close monitoring of ventilator screens by clinicians or through automated algorithms. However, detecting complex patient-ventilator interactions (CP-VI), consisting of changes in the respiratory rate and/or clusters of asynchronies, is a challenge. Sample Entropy (*SE*) of airway flow (*SE*-Flow) and airway pressure (*SE*-Paw) waveforms obtained from 27 critically ill patients was used to develop and validate an automated algorithm for detecting CP-VI. The algorithm’s performance was compared versus the gold standard (the ventilator’s waveform recordings for CP-VI were scored visually by three experts; Fleiss’ kappa = 0.90 (0.87–0.93)). A repeated holdout cross-validation procedure using the Matthews correlation coefficient (MCC) as a measure of effectiveness was used for optimization of different combinations of *SE* settings (embedding dimension, *m*, and tolerance value, *r*), derived *SE* features (mean and maximum values), and the thresholds of change (*Th*) from patient’s own baseline *SE* value. The most accurate results were obtained using the maximum values of *SE*-Flow (*m* = 2, *r* = 0.2, *Th* = 25%) and *SE*-Paw (*m* = 4, *r* = 0.2, *Th* = 30%) which report MCCs of 0.85 (0.78–0.86) and 0.78 (0.78–0.85), and accuracies of 0.93 (0.89–0.93) and 0.89 (0.89–0.93), respectively. This approach promises an improvement in the accurate detection of CP-VI, and future study of their clinical implications.

## Introduction

Invasive mechanical ventilation (MV) is a life-support measure administered to patients who cannot breathe on their own. Patient-ventilator asynchronies occur when there is a mismatch between the ventilator’s setting and patient’s breathing pattern. Recent studies have emphasized the impact of asynchronies upon clinical outcomes^1–5^, focusing on the incidence of specific subtypes of asynchronies or on the asynchrony index, and also on their distribution over time given that they occur in clusters within prolonged uneventful periods^1–6^. Importantly, in most of these studies ventilator’s waveforms were analysed visually^2,5,7,8^; only a few analyses have been based on automated algorithms^1,4,6,9,10^ or, more recently, on machine learning algorithms incorporating not only ventilator waveforms but also clinical data^11^.

Asynchronies are difficult to define when supported only by visual assessment carried out by inexperienced personnel, since different types may develop in a short time period or may even overlap with each other. Furthermore, asynchronies, which are by nature time-limited and transient, lead to patient distress, impede the ventilator’s effectiveness in decreasing the work of breathing, increase the time on mechanical ventilation and have a negative impact on outcome^1,2,4,7,12^. Additionally, sometimes patient’s drive only becomes evident due to an increase in the respiratory rate itself^13–16^, which, given its irregular and complex behaviour, may be overestimated by visual observation or dedicated algorithms. Therefore, it would be extremely useful to have access to a method for assessing irregularity and complexity which could detect Complex Patient-Ventilator interactions (CP-VI), including not just asynchronies of any kind but also changes in the respiratory rate, in an automated, non-invasive and personalized fashion.

Normal physiological data are non-linear^17^. The complex behavior of a non-linear system cannot be characterized by the sum of its inputs, and the study of these systems requires methods that take into account the non-linear physiological response to a given stimulus. These methods could provide insights into organ-system interconnectivity, regulatory control, and complexity in time series during disease^17–19^.

Entropy is a non-linear method derived from the theory of complex systems which measures the randomness and predictability of stochastic processes. Various types of entropy have been used in clinical monitoring^20–22^. Sample Entropy (*SE*) is a measure of complexity and regularity, defined as the negative natural logarithm of the conditional probability that two sequences similar for *m* points will remain similar at the next point, where self-matching is not included^23^. Thus, a lower *SE* value indicates more self-similarity in a time series.

*SE* has proved to be an effective tool for investigating different types of time series data derived from various biological conditions in the human body. Examples of these conditions include the activation of inspiratory muscles in COPD patients^24,25^, the analysis of atrial fibrillation on electrocardiograms^26^, background electroencephalograms in Alzheimer’s patients^27^, heart rate variability^28,29^, human postural sway^29^ and seizure termination during electroconvulsive therapy^30^.

Interestingly, only a few entropy approaches have been applied in the respiratory system to study breath-to-breath variability and its components as predictors of successful separation from MV during spontaneous breathing trials (SBT)^19,31–34^. Breath-to-breath approaches suggest that increased irregularity of the respiratory system may be a marker of pulmonary health^19^ and may serve as a weaning predictor^32–35^, opening up the possibility that a certain degree of irregularity may be normal^3,36^. However, these studies rely on the detection of the appropriate respiratory cycle. Hence, the performance of automated algorithms in breathing cycle detection may be jeopardized when transient asynchronies occur during patient-ventilator interaction or even overlap with each other. In this respect, other authors have applied the *SE* to the entire signal, as is the case of Sá et al.^37^ who evaluated the respiratory changes by applying *SE* upon the entire airway flow signal providing an early and sensitive functional indicator of interstitial asbestosis.

We hypothesized that analyzing transient complexity of CP-VI may provide clinically relevant information during MV. Therefore, we sought to develop and validate a non-invasive method based on *SE* measurement using the entire airway pressure (Paw) and airway flow (Flow) waveforms to detect CP-VI, defined as the occurrence of asynchronies and changes in the respiratory rate.

## Methods

### Defining complex patient ventilator interactions

**Figure 1:**
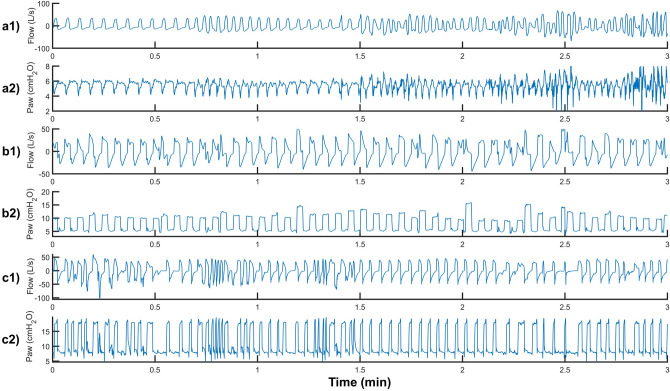
Tracing of Flow and Paw from three different patients. (**a**) Continuous Positive Airway Pressure (CPAP) of 6 cmH_2_O. (**b**) Pressure assist-control ventilation (PCV) with pressure of 10 cmH_2_O, (**c**) PSV with a pressure support of 10 cmH_2_O and PEEP of 8 cmH_2_O. In (**a1**) and (**a2**), Complex Patient-Ventilator Interactions (CP-VI) consists of an increase in respiratory rate > 50%; in (**b1)** and (**b2**), it consists of > 30% asynchronies (ineffective expiratory effort, double cycling, premature cycling, prolonged cycling, and/or reverse triggering) in the 3-min period; and in (**c1**) and (**c2**) it consists of a combination of change in the respiratory rate and asynchronies.

We defined CP-VI as a > 50% change in the respiratory rate^13,35,38,39^ and/or > 30% asynchronous breaths of any type (ineffective expiratory efforts, double cycling, premature cycling, prolonged cycling, or reverse triggering) over a 3-min period. A recent study found that 38% of mechanically ventilated patients had clusters of ≥ 30 ineffective expiratory efforts in a 3-min period (i.e., ≥ 50% of all breaths in a patient with a respiratory rate of 20 breaths per minute), and that the median duration of these clusters was 20 min^4^. Another study found that 59.7% of patients had clusters in which > 10% of all breaths in a 3-min period were double cycled, with a mean cluster duration of 15.5 min^6^. Figure 1 shows a representative example of different CP-VIs consisting of increased respiratory rate, asynchronies, or a combination of these phenomena.

### Data acquisition and data analysis

The Better Care system (Better Care, Barcelona, Spain. US patent No. 12/538,940) continuously records Paw and Flow signals at a sample frequency of 200 Hz from intubation to liberation from MV^9^. Better Care uses drivers specifically designed to interact with output signals from mechanical ventilators and bedside monitors rather than directly with patients, synchronizing recorded signals and storing them for further analysis. We used MATLAB (The MathWorks, Inc., vR2018b, Natick, MA, USA) for signal processing, data analysis, and visual assessment. Signals were decimated at a sampling rate of 40 Hz before entropy calculation.

### Study population

The findings presented in this paper represents an ancillary analysis on an ongoing clinical study (ENTROPY-ICU, ClincalTrials.gov NCT04128124) designed to assess the feasibility of using *SE* to identify CP-VI during MV. Data from 27 patients were obtained from an ongoing database at two centers in Spain. The database was constructed prospectively for the development of a connectivity platform (Better Care) to interoperate signals from different ventilators and monitors and subsequently compute algorithms for diagnosing patient-ventilator asynchronies (ClinicalTrial.gov, NCT03451461). The Comitè d’Ètica d’Investigació amb medicaments at the Corporació Sanitària Parc Taulí and the Clinical Research Ethics Committee of Fundació Unió Catalana d’Hospitals approved the database and the study protocol. The need for informed consent was waived because the current study was an ancillary analysis with anonymized data. The guidelines followed in this study were according to the applicable Spanish regulations (Biomedical Research Law 14/2007). This type of study must be evaluated and approved by at least one Institutional Review Board (IRB). Parc Taulí’s IRB approved this study to be carried out in all participating centers. The IRB approved the study allowing it to be carried out without the explicit request of informed consent from each participant given that it is a study with retrospective data. Spanish regulations allow studies to be carried out with this condition as long as they are approved by an IRB.

The *SE* analysis was performed on the complete set of Flow and Paw data collected during the two hours before self-extubation. Self-extubations, defined as extubations performed by the patient himself, are included in unplanned extubations but its mechanisms differ from accidental extubations^40^. Clinical and demographic data were obtained from medical charts (Table 1).

**Table 1:** Patient characteristics. APACHE II: Acute Physiology and Chronic Health Evaluation.

Clinical and demographic data of patient	
Sex	
Male, n (%)	22 (81.5%)
Female, n (%)	5 (18.5%)
Mean age (range), in years	63.8 (57–72)
APACHE II at admission	16.7 (9–22)
Mean ICU–LOS (range), in days	18.7 (7.5–27)
Mean hospital–LOS (range), in days	34 (15.5–41)
Reason for MV, n (%)	
Respiratory insufficiency	9 (33.3%)
Sepsis/septic shock	10 (37%)
Altered consciousness	3 (11.1%)
Others	7 (25.9%)
Use of sedatives (%)	71.4%
RASS	0.6 ± 1.7

*ICU* intensive care unit, *LOS* length of stay, *MV* mechanical ventilation, *RASS* Richmond Agitation-Sedation Scale.

### Visual validation of CP-VI

Experts’ visual assessment was considered the gold standard. Three critical care physicians with extensive experience in analyzing ventilator waveforms visually reviewed 92 15-min-long segments of Flow and Paw recordings from the two-hour period immediately before self-extubation. The 15-min window was selected based on two previous studies evaluating clusters of asynchronies, in which mean cluster duration was 15.5 and 20 min respectively^6,10^. An expert in MV selected the segments to ensure a balanced proportion of different ventilation modes (grouped into pressure support ventilation (PSV) or assist-control ventilation (ACV) modes, comprising volume assist-control and pressure assist-control ventilation) and of segments with and without CP-VIs. Every patient contributed both CP-VI and non-CP-VI segments with at least one 15-min segment of each type; however, some patients contributed more segments than others. In order to ensure that the most valuable CP-VI events were not missed, all the 15-min segments immediately preceding self-extubation were included. To ensure masking of the scorers, Flow and Paw tracings were randomly ordered in MATLAB prior to visual analysis. To standardize scoring criteria, scorers were provided with written descriptions of the characteristics of CP-VI before visual analysis. Scorers were asked to determine whether CP-VI were present in each segment. No time limitations were imposed.

### Sample entropy

*SE* is a non-linear technique that measures the randomness of a series of data^23^. Compared to other approaches, *SE*’s main advantage is that it provides consistent results even in short and noisy medical time series^19,23^. To calculate *SE*, three parameters are necessary: the embedding dimension, *m* (a positive integer); the tolerance value or similarity criterion, *r* (a positive real number); and the total length of the series, *N*. Briefly, *SE* is defined as the negative logarithm of the conditional probability that two sequences of patterns of *m* consecutive samples that are similar to each other within a tolerance *r* will remain similar when one consecutive sample is added ($m + 1$), excluding self-matches. *SE* is calculated as follows^23^:

Given a time series of N samples $\left\{ {x\left( n \right) = x\left( 1 \right),x\left( 2 \right), \ldots ,x\left( N \right)} \right\}$, a subset of $N - m + 1$, overlapping vectors $X_{m} \left( i \right)$ of length $m$ are defined:

1. Form *m* vectors defined by $X_{m} \left( i \right) = \left[ {x\left( i \right),x\left( {i + 1} \right), \ldots ,x\left( {i + m - 1} \right)} \right],{ }i = 1,2, \ldots ,N - m + 1$. These represent $m$ consecutive $x$ values.
2. Then, define Chebyshev distance between vectors ${ }X_{m} \left( i \right)$ and $X_{m} \left( j \right)$, i.e., the maximum absolute difference between their scalar components:
  1 $$d\left[ {X_{m} \left( i \right),X_{m} \left( j \right)} \right] = \begin{array}{*{20}c} {max} \\ {k = 0, \ldots ,m - 1} \\ \end{array} \left[ {\left| {x\left( {i + k} \right) - x\left( {j + k} \right)} \right|} \right].$$
3. For a given $X_{m} \left( i \right)$, count the number of j $\left( {1 \le j \ge N - m,i \ne j} \right)$, denoted as $B_{i} \left( r \right)$, such that the distance between $X_{m} \left( i \right)$ and $X_{m} \left( j \right)$ is less than or equal to a threshold $r$.
  2 $$\begin{gathered} \hfill \\ B_{i}^{m} \left( r \right) = \frac{{B_{i} \left( r \right)}}{N - m - 1}, \\ \text{Then, for}\, 1 \le i \ge N - m, \hfill \\ \end{gathered}$$
4. Defined $$B^{m} \left( r \right)$$ as
  3 $$B^{m} = \frac{1}{N - m}\mathop \sum \limits_{i = 1}^{N - m} B_{i}^{m} \left( r \right)$$
5. This previous procedure is repeated, increasing the dimension to $m + 1$ to calculate $A_{i} \left( r \right)$ as the number of $X_{m + 1} \left( i \right)$ within $r$ of $X_{m + 1} \left( j \right)$, where $j$ ranges from 1 to $N - m\left( {i \ne j} \right)$. Then,${ }A_{i}^{m} \left( r \right)$ is defined as:
  4 $$A_{i}^{m} \left( r \right) = \frac{{A_{i} \left( r \right)}}{N - m - 1}$$
6. Set $$A^{m} \left( r \right)$$ as
  5 $$A^{m} = \frac{1}{N - m}\mathop \sum \limits_{i = 1}^{N - m} A_{i}^{m} \left( r \right)$$

Thus, $B^{m} \left( r \right)$ is the probability that two sequences will match for $m$ samples, whereas $A^{m} \left( r \right)$ is the probability that two sequences will match for $m + 1$ samples. Finally, sample entropy is then defined as

6 $$SE\left( {m,{ }r} \right) = \mathop {\lim }\limits_{N \to \infty } \left\{ { - ln\left[ {\frac{{A^{m} \left( r \right)}}{{B^{m} \left( r \right)}}} \right]} \right\}{ }$$

which is estimated by the statistic:

7 $$SE\left( {m, r, N} \right) = - ln\left( {\frac{{A^{m} \left( r \right)}}{{B^{m} \left( r \right)}}} \right)$$

The *m* parameter is generally taken as 2, while the *r* parameter normally ranges between 0.1 and 0.25 times the standard deviation (SD) of the segment analyzed of length *N*. In this study, *SE* was calculated over the Flow (*SE*-Flow) and Paw (*SE*-Paw) signals using a 30-s sliding window (*N* = 1,200 samples) with 50% overlap. *SE* was explored using *m* from 1 to 20 and with *r* values equal to 0.1, 0.2, 0.3, and 0.4 times the SD of each sliding window. To reduce noise and to increase the consistency of the results, we applied an 8-period-long exponential moving average filter to the *SE* series.

### Automatic CP-VI detection

**Figure 2:**
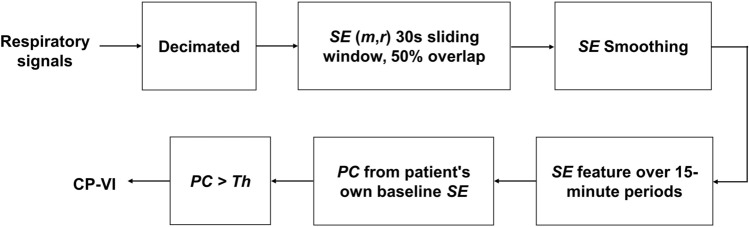
Automatic CP-VI detection. Respiratory signals (Flow and Paw) are decimated at a sampling rate of 40 Hz. Sample entropy (*SE*) is calculated for different values of the embedding dimension (*m*) and tolerance (*r*). An 8-period-long exponential moving average is used to reduce noise and to increase the consistency of the *SE* results. Two *SE* features are determined for each 15-min period: the mean value and the maximum value. The percentage of change (*PC*) from the patient's own baseline value is calculated for each *SE* setting. When *PC* exceeds a determined threshold (*Th*), the period is considered to contain a CP-VI event. An optimization procedure is required to select the values of *m*, *r*, *Th*, respiratory signal, and *SE* feature that yield the most robust estimations of CP-VI.

We devised an automated algorithm based on *SE* to detect CP-VI events (European patent application number EP19383116). Figure 2 summarizes the algorithm in a flowchart. Detection of a CP-VI depends on whether the percentage of change (*PC*) in *SE* with respect to the patient's own *SE* baseline value during the 15-min period is greater than a predefined threshold of change (*Th)*. We calculated *PC* for *SE*-Flow and *SE*-Paw in each 15-min period in two ways, using the following derived features (the mean *SE* value [*SE*-Flow_mean_ and *SE*-Paw_mean_], and the maximum *SE* value [*SE*-Flow_max_ and *SE*-Paw_max_]), applying different values of *Th* (15%, 20%, 25%, 30%, 35%, 40%, 45%, and 50%). We hypothesized that *SE* values would be higher in periods with CP-VI than in periods with regular patient-ventilator interactions. Periods were considered to contain a CP-VI event when *PC* exceeded the *Th*. The optimal *Th* for CP-VI detection was selected during the *SE* setting optimization procedure (explained below).

Keim-Malpas^41^ recently proposed that alert thresholds derived from continuous analytic monitoring should be based on the degree of change from the patient’s own baseline, rather than on general cutoff thresholds. In our study there was no single baseline value common to all patients; each patient had their own baseline.

The baseline value of each *SE* feature was initialized with the value calculated in the first 15-min period. This value was updated with each new 15-min segment if the *SE* feature of the new one was lower than the current baseline.

### Statistical analysis

Fleiss’ kappa coefficient was used to assess the reliability of agreement among scorers for visual assessment^42^. The automated CP-VI detection algorithm was applied over the *SE* series derived from the same Flow and Paw tracings previously used for visual assessment. To evaluate the performance of the automated algorithm with respect to the gold standard visual assessment, we calculated sensitivity, specificity, positive and negative predictive values (PPV and NPV respectively), accuracy, and the Matthews correlation coefficient (MCC)^43^. Widely used in biomedical research, the MCC is considered a balanced measure of the confusion matrix of true and false positives and negatives^44–46^. Calculation of the MCC is based on all four elements of the confusion matrix: true positive (TP), true negative (TN), false positive (FP), and false negative (FN) values, as follows:

8 $$MCC = \frac{{TP{*}TN - FP{*}FN}}{{\sqrt {\left( {TP + FP} \right){*}\left( {TP + FN} \right){*}\left( {TN + FP} \right){*}\left( {TN + FN} \right)} }}$$

MCC values can range from − 1 to + 1. An MCC value of − 1 suggests perfect disagreement between the predictions and the gold standard, and a value of 1 suggests perfect agreement between the predictions and the gold standard; a value of 0 indicates that the prediction is no better than random. The MCC index was used as the measure of effectiveness during the process to optimize *SE* settings so as to achieve the most robust CP-VI estimation.

### Optimization procedure (selection of *m*, *r*, and *Th*)

In entropy studies, determining the optimal settings to robustly extract the randomness of a series of data is an important step^47,48^. To select the optimal settings for the *SE* parameters *m* and *r* and the optimal *Th* for estimating CP-VI, we used a repeated holdout cross-validation method with the MCC as a measure of effectiveness.

**Figure 3:**
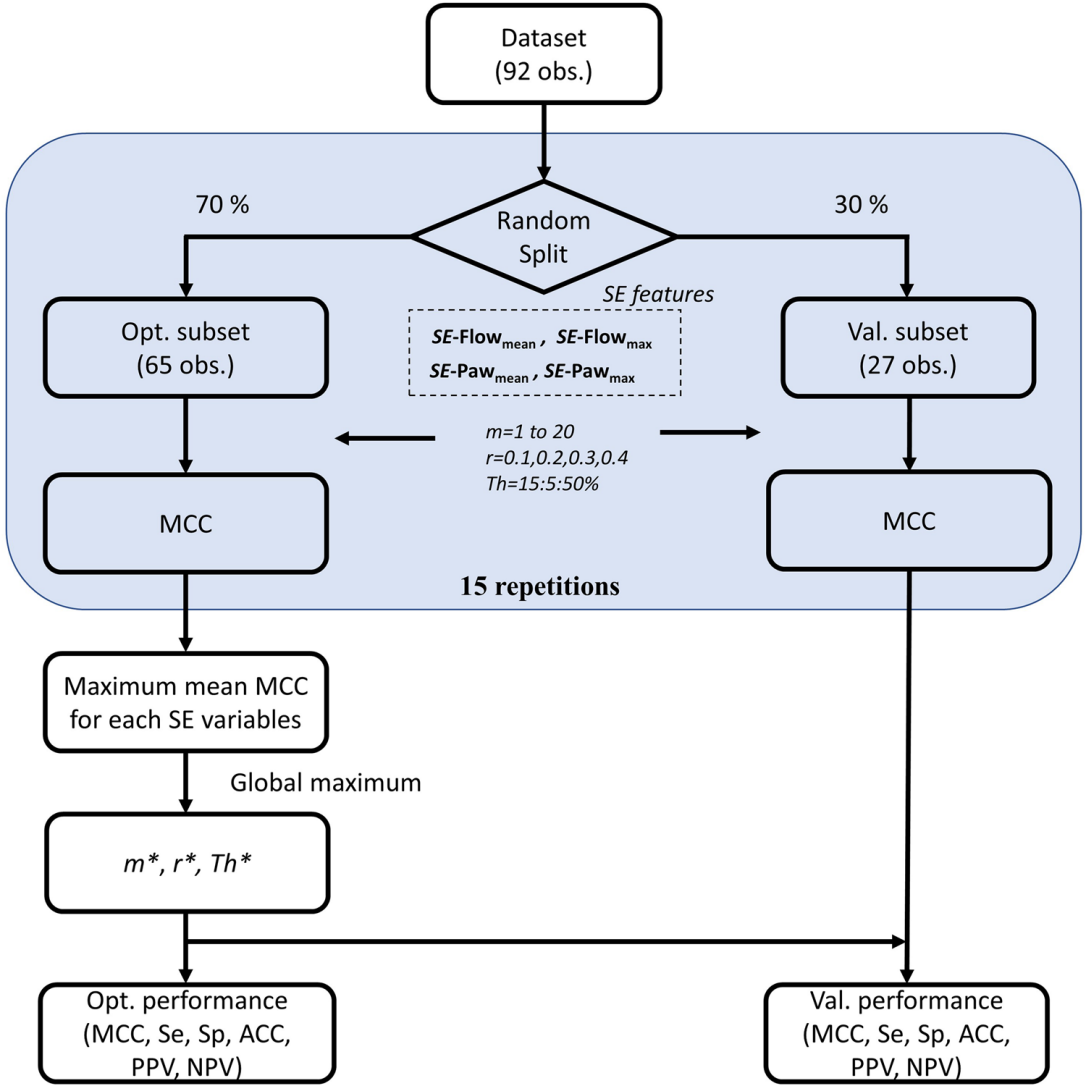
Flowchart for the optimization procedure and validation. Procedure to select the optimal sample entropy (*SE*) settings (*m* and *r*) and the threshold of change (*Th*) for each *SE* airway flow (Flow) and airway pressure (Paw) features (*SE*-Flow_mean_, *SE*-Flow_max_, *SE*-Paw_mean_, and *SE*-Paw_max_). The dataset visually validated by the experts was randomly divided into two subsets: optimization (Opt.) and validation (Val.). The optimization procedure was repeated a total of 15 times using different subsets (randomly selected each time). The global maximum mean value of the Matthews correlation coefficient (MCC) determined the optimal values of m*, r*, and Th* among all possible combinations and all *SE*-derived features. Finally, the mean values of the measures of accuracy were computed for the optimal combination of parameters in both the optimization and the validation subsets.

Figure 3 depicts the steps involved in the optimization and the validation procedure. Once the experts had visually validated the set of 92 observations, it was randomly divided into two subsets: 70% of the data for optimization and the remaining 30% of the data for validation. This optimization procedure was repeated a total of 15 times using different subsets (randomly selected each time) to capture as much relevant information as possible and to minimize the potential bias resulting from fitting the settings on a single partition. The MCC metric was computed for all combinations of *m*, *r*, and *Th* for each repetition. Finally, the maximum mean MCC value determined the optimal combination of *SE* settings and *Th* among all possible combinations. The optimization procedure was individually applied to the features derived from *SE*-Flow (*SE*-Flow_mean_, *SE*-Flow_max_) and *SE*-Paw (*SE*-Paw_mean_, *SE*-Paw_max_) in order to determine the respiratory signal and features that best reflect CP-VI.

In addition, a sensitivity analysis by using a small grid search of *r* values (step = 0.01) around the optimal value in the best features derived from *SE*-Flow and *SE*-Paw was performed to compare regions of confidence and to investigate whether the selected *r* value is a robust local maximum.

To assess the robustness of the optimization procedure, we computed the medians and interquartile ranges of all measures of performance (MCC, sensitivity, specificity, accuracy, PPV, and NPV) considering the optimal combination for both the optimization and validation subsets.

## Results

### Visual CP-VI analysis by experts

The experts visually assessed a total of 92 periods: 45 periods of PSV (22 with CP-VI and 23 without) and 47 periods of ACV (24 with CP-VI and 23 without). Fleiss’ kappa for inter-rater agreement was 0.90 (0.87–0.93), indicating almost perfect agreement.

### Detecting CP-VI with *SE*

**Figure 4:**
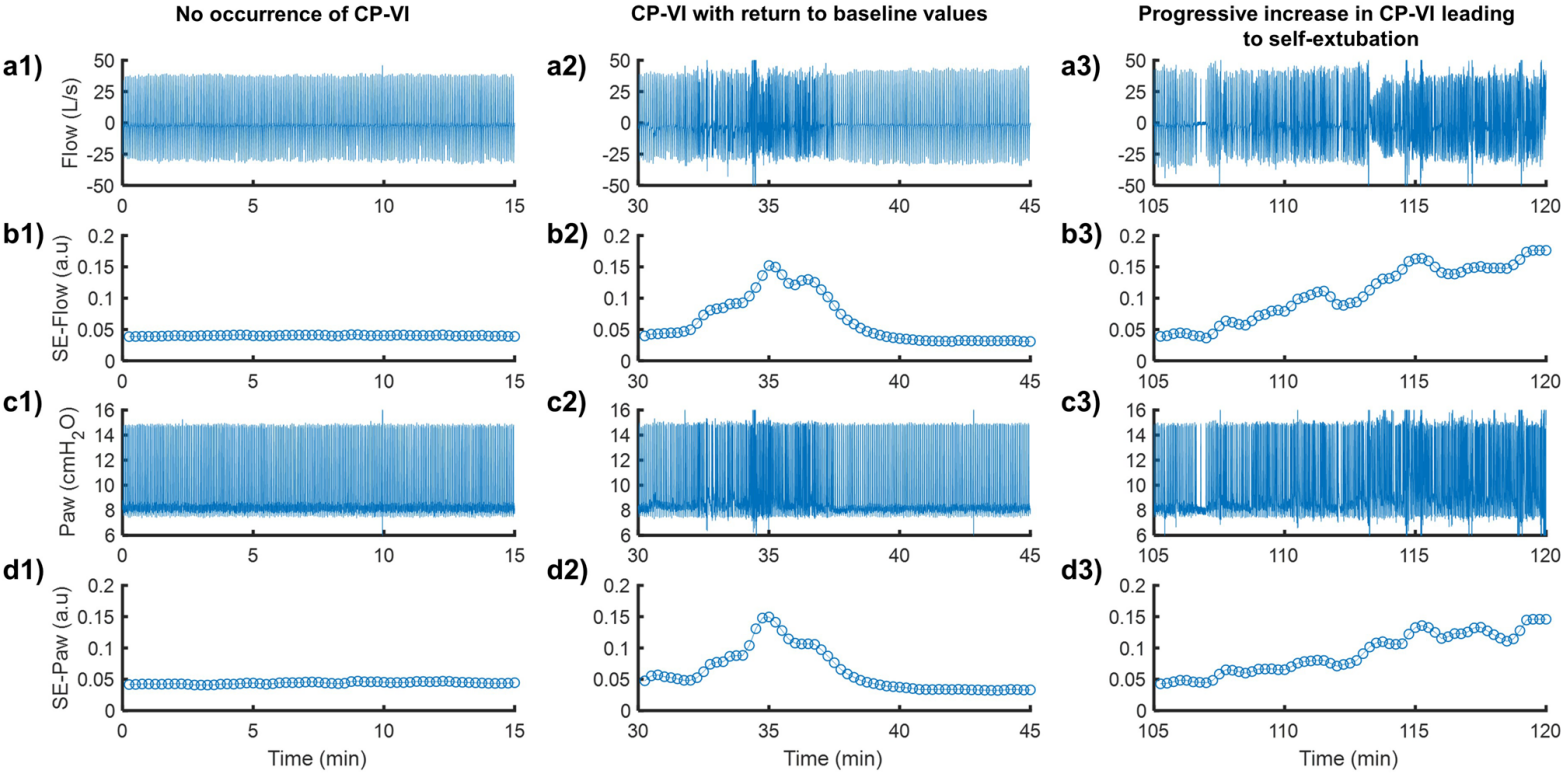
Representative examples of ventilator signals for airway flow (Flow) (**a1**–**a3**) and airway pressure (Paw) (**c1**–**c3**) recorded over 15-min periods in the two hours prior to self-extubation, together with the sample entropy (*SE*) tracings derived from Flow (**b1**–**b3**) and Paw (**d1**–**d3**). Both, *SE*-Flow and *SE*-Paw were calculated with *r* = 0.2 × SD of each overlapping 30-s-long sliding window, by using different values of *m* equal to 2 and 4, respectively. Three 15-min periods are represented, corresponding to (1) no occurrence of CP-VI (left panel), (2) occurrence of CP-VI that returned to baseline values (middle panel), and (3) progressive increase in CP-VI leading to self-extubation. *SE* is highly sensitive to changes in irregularity during MV.

The exponential moving average filter reduced the noise in *SE* series and generated a smoothed *SE* version suitable for detecting CP-VI (see Supplementary Methods and Supplementary Fig. S1). Figure 4 shows representative examples of respiratory signal tracings with the corresponding *SE*-Flow (*m* = 2 and *r* = 0.2) and *SE*-Paw (*m* = 4 and *r* = 0.2) tracings. *SE* was highly sensitive to changes in the irregularity of the respiratory pattern occurring during ventilation.

### Optimization of *SE* settings, *Th* detection using a repeated holdout cross-validation procedure

**Figure 5:**
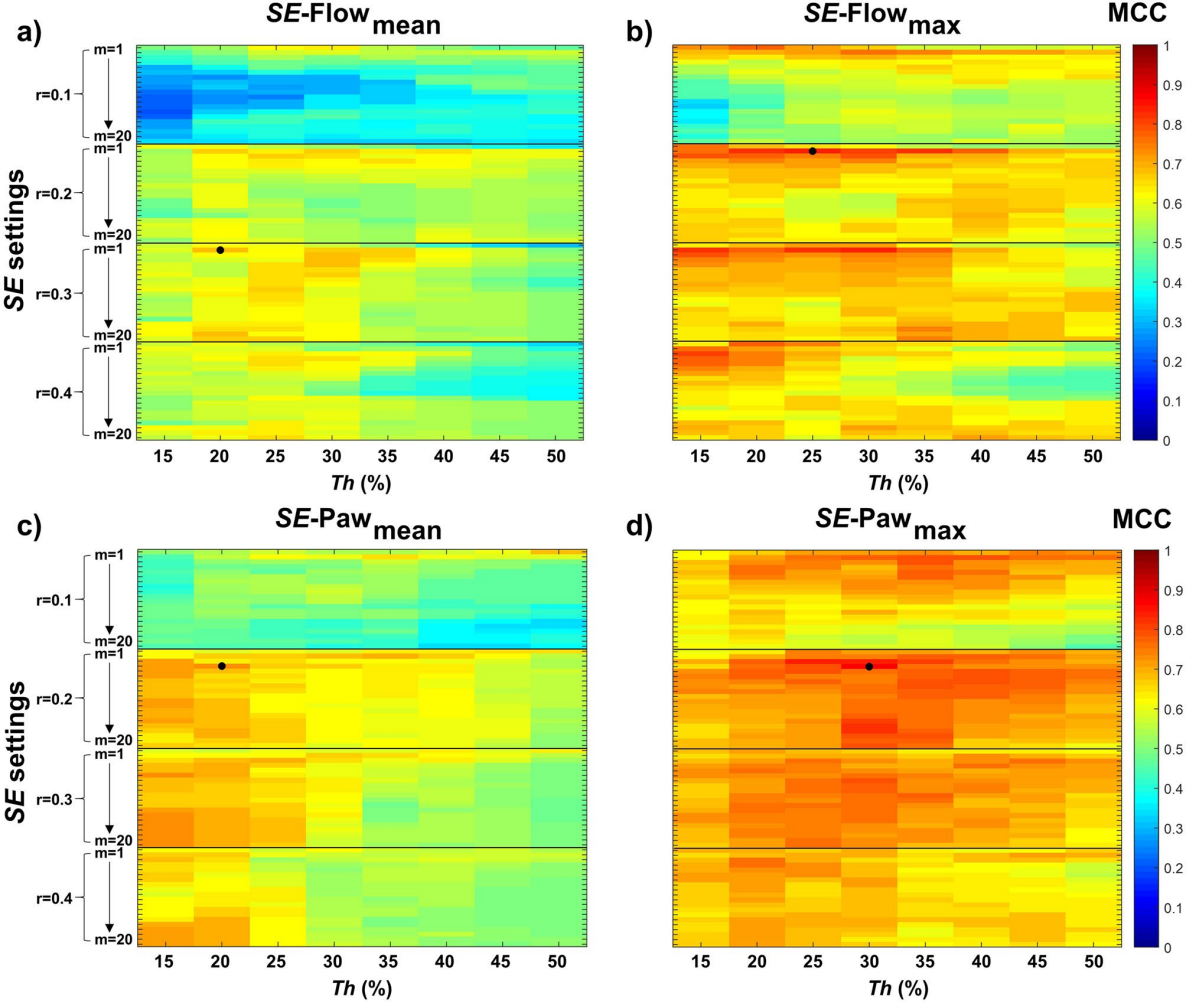
Repeated holdout cross-validation process for optimization of sample entropy (*SE*) settings and threshold (*Th*) for the detection of complex patient-ventilator interactions. We calculated the mean Matthews correlation coefficient (MCC) for each derived feature analyzed and each combination of *m* (1 to 20), *r* (0.1, 0.2, 0.3, and 0.4 times the SD of each sliding window), and *Th* (15%, 20%, 25%, 30%, 35%, 40%, 45%, and 50%) a total of 15 times using different randomly selected subsets. The upper panels show the results of the optimization process for *SE*-airway flow (*SE*-Flow), and the lower panels show the results for *SE*-airway pressure (*SE*-Paw). The color bar in each subplot shows the mean MCC scale, where values near 1 indicate more robust and consistent results. The MCC was positive in all cases. The black dot in each subplot indicates the combination that yielded the maximum mean MCC.

**Figure 6:**
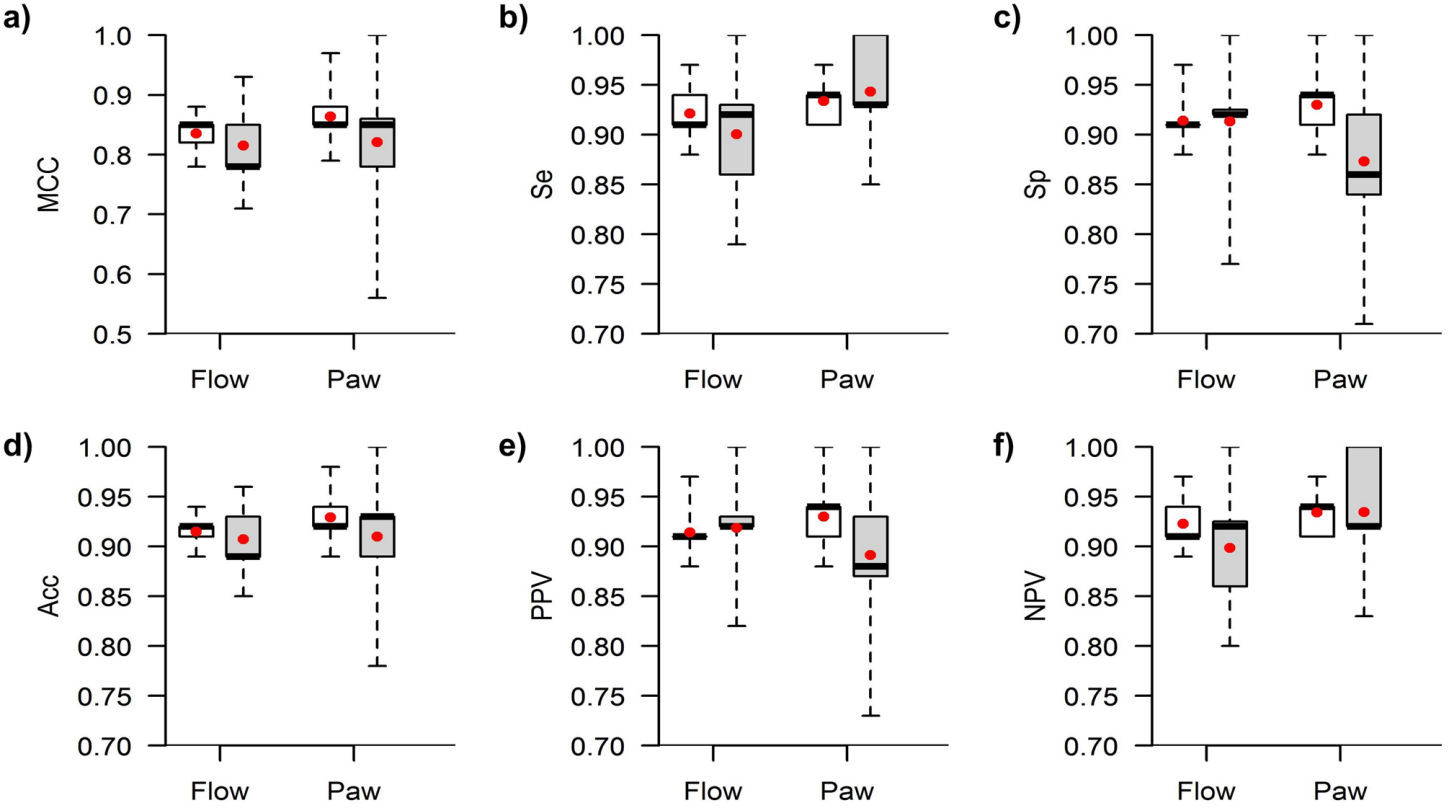
Performance statistics for *SE*-Flow_max_25 (*m* = 2 and *r* = 0.2) and *SE*-Paw_max_30 (*m* = 4 and *r* = 0.2) for detecting CP-VI. Boxplots of (**a**) Matthews correlation coefficient (MCC), (**b**) sensitivity (Se), (**c**) specificity (Sp), (**d**) accuracy (Acc), (**e**) positive predictive value (PPV), and (**f**) negative predictive value (NPV) from 15 repetitions during optimization (white) and 15 repetitions during validation (gray). The red dot represents the mean value.

Figure 5 shows the procedure used to optimize *SE* settings and *Th* for CP-VI detection. We calculated the mean MCC value for each combination of *m*, *r*, and *Th* for all derived features analyzed (*SE*-Flow_mean_, *SE*-Flow_max_, *SE*-Paw_mean_, and *SE*-Paw_max_). In general, *SE*-Paw features exhibit much less sensitivity to *m* parameter selection than *SE*-Flow features. *SE*-Flow_max_ and *SE*-Paw_max_ features yielded the highest mean MCC values. The highest MCC values for *SE*-Flow_max_ were found for values of *m* = 2, *r* equal to 0.2 and 0.3, and *Th* between 20 and 35%, whereas for *SE*-Paw_max_ were found for values of *m* equal to 3 and 4, *r* = 0.2, and *Th* between 25 and 30%. The optimal *SE* settings for *SE*-Flow_max_ were *m* = 2, *r* = 0.2, and *Th* = 25%, and for *SE*-Paw_max_ *m* = 4, *r* = 0.2, and *Th* = 30%. As regards the optimal *SE* settings, *SE*-Flow_max_ at *Th* = 25% (*SE*-Flow_max_25) yielded the highest mean MCC value (0.84) and *SE*-Paw_max_ at *Th* = 30% (*SE*-Flow_max_30) yielded the highest mean MCC value (0.86). Both *SE*-Flow_max_25 and *SE*-Paw_max_30 yielded their highest MCC values in 13 of the 15 repetitions. The sensitivity analysis conducted for the *SE*-Paw_max_ and *SE*-Flow_max_ features around the optimal value of *r* = 0.2 is shown in Supplementary Figure S3. Once we had determined the settings that best detected CP-VI, we evaluated the performance of the algorithm in the 15 repetitions of the cross-validation procedure. Figure 6 displays the algorithm’s performance statistics. The median values of all the parameters observed in the optimization subset were slightly higher than those observed in the validation subset (Supplementary Table S1); this is a common consequence of the repeated holdout cross-validation process. The performance of *SE*-Flow_max_25 and *SE*-Paw_max_30 stratified by ventilator modality (grouped into pressure support ventilation and assist-control ventilation modes) is shown in Supplementary Table S2.

For comparative purposes, we also carried out the procedure for optimizing *SE* settings and *Th* over the unfiltered *SE* series. The Supplementary Methods and the Supplementary Figure S2 show the results obtained in this case.

## Discussion

Our automatic algorithm for detecting CP-VI from ventilator signals proved highly sensitive and specific in individual patients. Using non-linear analysis of *SE* to measure irregularity and randomness in the entire set of physiological Flow and Paw signals, the algorithm compared data from different periods in each patient’s interaction with the ventilator to detect CP-VI. In our analyses the maximum changes of *SE* in both Flow and Paw signals yielded the most accurate results at different thresholds and settings. The most accurate results for *SE*-Flow_max_ were obtained with a threshold of change of 25% with *m* = 2, *r* = 0.2, and for *SE*-Paw_max_ with a threshold of change of 30% with *m* = 4 and *r* = 0.2.

The recognition of the hidden information contained in physiological time series draws attention to the extraordinary complexity of physiological systems^49^. Several non-linear techniques have been developed to study the irregularity and complexity of these physiomarkers^18,23,50–53^. Previous studies have used methods based on approximate entropy and sample entropy using breath-to-breath variability and derived indices^19,23,32–34^, which relies on the detection of the appropriate respiratory cycle.

The main advantage of our approach is that it does not require the detection of each single breathing cycle to measure irregularity in Flow and Paw waveforms and thus identify the development of a CP-VI. This approach makes a fundamentally different assumption about where complexity occurs in the physical signal, focusing on transient Flow and Paw complexity rather than breath-to-breath complexity in order to accurately identify changes in the respiratory rate and asynchronies which by their nature are transient and time-limited.

To our knowledge, no recommendations are currently available for the estimation of respiratory dynamics by applying an entropy approach to the entire dataset of Flow and Paw tracings during MV^23,52^. Recently, Sá et al.^37^, used *SE* estimation upon entire Flow signal without optimized parameters. Thus, one important contribution of our study is the description of a set of optimization and validation procedures based on a repeated holdout cross-validation method used in machine-learning models, which we used to obtain the optimal *m*, *r* and *Th* values. Ensuring the robustness of the validation procedure.

Our study also applied a personalized threshold to determine the occurrence of a CP-VI event based on a proportional change from the patient’s own baseline value, which is continuously updated. Continuous predictive analytics monitoring achieves early detection of changes in status over time in previously stable patients. Keim-Malpas et al.^41^ recently suggested that an absolute threshold of change from baseline values may not be clinically significant in real-world settings and could lead to a high rate of false-positives in patients with high baseline values^54^. In our study, thresholds of change of 25% and 30% from *SE*-Flow_max_ and *SE*-Paw_max_ respectively, proved to be the most accurate for CP-VI detection. The optimization procedure found that *r* = 0.2 is suitable for detecting CP-VI events using *SE*-Flow_max_ (*m* = 2, *Th* = 25%) or *SE*-Paw_max_ (*m* = 4, *Th* = 30%) features. Additionally, the sensitivity analysis indicates that *r* = 0.2 proved to be a more robust local maximum for *SE*-Flow_max_ feature. This might suggest that the algorithm predictions seems to be not influenced by small changes in underlying unknown parameters (i.e., different dataset, different measurement equipment or ventilator waveforms) when using *SE*-Flow_max_ (*m* = 2, *r* = 0.2, *Th* = 25%), and therefore, could be a more suitable feature than *SE*-Paw_max_ (*m* = 4, *r* = 0.2, *Th* = 30%).

Interestingly, both *SE*-Flow_max_25 and *SE*-Paw_max_30 performed well in detecting CP-VI in Assist-Control Ventilation, while *SE*-Flow_max_25 performed slightly better than *SE*-Paw_max_30 in Pressure Support Ventilation mode. The reason for the latter finding may be that during PSV the pressure is constant, and it is the flow waveform that exhibits more changes in accordance with patient’s demand and the mechanical properties of the diseased lung. However, due to the small sample size these sub-analysis results should be interpreted with care, and further research is needed.

Our study has several limitations. First, our algorithm responds to changes in the respiratory rate based on transient changes of Flow and Paw waveforms detected by *SE*, but not on inspiratory effort. This means that respiratory drive, the intensity of the neural output from the respiratory center that regulates the magnitude of inspiratory effort^55^, may not have been fully assessed^15,56,57^. Unfortunately, although many techniques have been proposed^55,58–60^ none have been implemented at the bedside to monitor drive and effort. Our proposed algorithm does not include measurements of effort; nevertheless, whenever a diaphragmatic contraction occurs unassisted by the ventilator, and an asynchrony develops our algorithm is able to detect it.

Second, although our method does not rely on the detection of breathing cycles to measure irregularity and is based on changes in *SE* of Flow and Paw waveforms, none of the features deriving from breath-to-breath variability were considered. Therefore, their potential importance in detecting CP-VI is yet to be assessed.

Third, while the dataset used for the repeated hold out cross-validation method was paired between segments with and without CP-VI, most of them were from tracings of patients who self-extubate, in whom the occurrence of events of poor patient-ventilator interactions is highly unpredictable. For that reason, the clinical meaning of CP-VI in critically ill patients is yet to be determined and requires more research. Additionally, in the current study we have only examined *SE*, and other promising measures of entropy may also provide adequate diagnostic tool. For instance, multiscale entropy analysis^61,62^, Fuzzy approximate entropy^63^, conditional entropy^64^ and distribution entropy^65^ could be others potentially useful entropy measures to be investigated.

Finally, we did not analyze data from proportional modes of MV. Thus, although it is tempting to speculate that ventilatory modes that adapt to patients’ efforts and variability might induce higher changes in *SE*, the performance of our algorithm in patients ventilated in these modes may differ substantially, and it should not be implemented in these modes until validated by future research.

## Conclusion

Our non-invasive method based on *SE* measurement of Paw and Flow is able to detect CP-VI, defined as the occurrence of transient asynchronies and changes in the respiratory rate, with high accuracy. Clinical relevance and usefulness of identifying Complex Patient-Ventilator Interactions in different clinical scenarios deserves to be explored.

## Supplementary information

Supplementary Information.

## Data Availability

The datasets generated and analyzed in the current study are available from the corresponding author on reasonable request.
