# Publisher Correction: Development and validation of a sample entropy-based method to identify complex patient-ventilator interactions during mechanical ventilation

**DOI:** 10.1038/s41598-020-75424-8

**Published:** 2020-11-09

**Authors:** Leonardo Sarlabous, José Aquino‑Esperanza, Rudys Magrans, Candelaria de Haro, Josefina López‑Aguilar, Carles Subirà, Montserrat Batlle, Montserrat Rué, Gemma Gomà, Ana Ochagavia, Rafael Fernández, Lluís Blanch

**Affiliations:** 1 Critical Care Center, Hospital Universitari Parc Taulí, Institut D’Investigació I Innovació Parc Taulí I3PT, Universitat Autònoma de Barcelona, Parc Taulí 1, 08208 Sabadell, Barcelona, Spain; 2 Biomedical Research Networking Center in Respiratory Disease (CIBERES), Instituto de Salud Carlos III, Madrid, Spain; 3 Facultat de Medicina, Universitat de Barcelona, Barcelona, Spain; 4 Biomedical Research Networking Center in Bioengineering, Biomaterials and Nanomedicine (CIBER‑BBN), Instituto de Salud Carlos III, Madrid, Spain; 5 BetterCare S.L, Sabadell, Spain; 6 Department of Intensive Care, Fundació Althaia, Universitat Internacional de Catalunya, Manresa, Spain; 7 Department of Basic Medical Sciences, Universitat de Lleida-IRBLLEIDA, Lleida, Spain

Correction to:* Scientific Reports* 10.1038/s41598-020-70814-4, published online 17 August 2020

This Article contains an order error in Equation 2,

“For a given X_m_(i), count the number of j (1 ≤ j ≥ N − m, i ≠ j), denoted as B_i_(r), such that the distance between X_m_(i) and X_m_(j) is less than or equal to a threshold r.”

$$ B_{i}^{m} \left( r \right) = \frac{{B_{i } \left( r \right)}}{N - m - 1}, $$

Then, for $$1 \le i \ge N - m,$$

should read:

“For a given X_m_(i), count the number of j (1 ≤ j ≥ N − m, i ≠ j), denoted as B_i_(r), such that the distance between X_m_(i) and X_m_(j) is less than or equal to a threshold r. Then, for $1 \le i \ge N - m,$”

$$ B_{i}^{m} \left( r \right) = \frac{{B_{i } \left( r \right)}}{N - m - 1}, $$

