# Supplementary material for: Development and validation of a sample entropy-based method to identify complex patient-ventilator interactions during mechanical ventilation
Source: Sci Rep. 2020 Aug 17;10:13911. doi: 10.1038/s41598-020-70814-4 (PMC7431581; doi:10.1038/s41598-020-70814-4)
Supplement: Supplementary file 1 — Supplementary Information. [file 41598_2020_70814_MOESM1_ESM.docx]

**Supplementary Material**

**Development and validation of a sample entropy-based method to identify complex patient-ventilator interactions during mechanical ventilation**

Leonardo Sarlabous^1,4^**^*^**, José Aquino-Esperanza^1,2,3*^, Rudys Magrans^5^, Candelaria de Haro^1,2^, Josefina López-Aguilar^1,2^, Carles Subirà^6^, Montserrat Batlle^6^, Montserrat Rué^7^, Gemma Gomà^1^, A. Ochagavia^1,2^, Rafael Fernández^2,6^, Lluís Blanch^1,2,5^.

* These authors contributed equally to this work.

^1^: Critical Care Center, Hospital Universitari Parc Taulí, Institut d’Investigació i Innovació Parc Taulí I3PT, Sabadell, Universitat Autònoma de Barcelona, Spain.

^2^: Biomedical Research Networking Center in Respiratory Disease (CIBERES), Instituto de Salud Carlos III, Madrid, Spain.

^3^: Universitat de Barcelona, Facultat de Medicina, Barcelona, Spain.

^4^: Biomedical Research Networking Center in Bioengineering, Biomaterials and Nanomedicine (CIBER-BBN), Instituto de Salud Carlos III, Madrid, Spain.

^5^: BetterCare S.L. Sabadell, Spain.

^6^: Department of Intensive Care, Fundació Althaia, Universitat Internacional de Catalunya, Manresa, Spain.

^7^: Department of Basic Medical Sciences, Universitat de Lleida-IRBLLEIDA, Lleida, Spain.

**Contents**

Supplementary Methods

Exponential moving average filter applied over the SE series

Optimization of *SE* settings and threshold detection using repeated holdout cross-validation over unfiltered *SE* series

Supplementary Figure S1

Supplementary Figure S2

Supplementary Figure S3

Supplementary Table S1

Supplementary Table S2

**Exponential moving average filter applied over the SE series**

To reduce noise and better expose consistency in Sample Entropy (*SE*) in respiratory signals from mechanically ventilated patients in whom Complex Patient-Ventilator Interactions (CP-VI) are clearly suspected, it is necessary to apply a smoothing step.

In this study, we used an 8-period-long exponential moving average (EMA) filter for this purpose. Figure S1 shows a representative example of unfiltered *SE* (red trace) and after applying the EMA filter (back trace) to airway flow (Flow) Flow (upper plot) and airway pressure (Paw) (lower plot) signals traces. The EMA significantly reduced the noise present on *SE* series, generating a smoothed *SE* version suitable for detecting CP-VI.

**Optimization of *SE* settings and threshold detection using repeated holdout cross-validation over unfiltered *SE* series**

Supplementary Figure S2 shows the procedure to optimize *SE* settings (*m* and *r*) and the threshold (*Th*) for the detection of CP-VI applied to the unfiltered *SE* series. In general, no significant changes were observed for *SE*-Flow_mean_, *SE*- and *SE*-Paw_mean_ with respect to those obtained when applying the EMA filter.

On the other hand, important changes were observed for *SE*-Flow_max_ and *SE*-Paw_max_. For *SE*-Paw_max_, the maximum values of the mean MCC were similar in some cases than those obtained after applying the EMA filter (see Figure 5 of manuscript). In this case, *Th* values were more concentrated towards higher values. Specifically, not filtering the *SE*-Paw signal requires using *Th* above 25%, being any value of *m* in combination with *r* higher than 0.1 suitable for estimating CP-VI. For *SE*-Flow_max_, the mean MCC values were generally lower than 0.8 after applying the EMA filter.

**Supplementary Figure S1.** Representative example of the effects of applying an 8-period-long exponential moving average filter over the *SE*-Flow (upper plot) and *SE-*Paw (lower plot) using *m*=3 and *r*=0.2 X standard deviation of each overlapping 30-second-long sliding window.

**Supplementary Figure S2.** Mean Matthews correlation coefficient (MCC) metric resulted from the repeated holdout cross-validation procedure to optimize the *SE* settings and the *Th* for CP-VI detection over **unfiltered *SE* series**. Each feature analysed is represented on an independent subplot (*SE*-Flow, top panels; *SE*-Paw, bottom panels), and for each one, the combination of *m* (1 to 20), *r* (equal to 0.1, 0.2, 0.3 and 0.4 times the SD of each sliding window), and *Th* (15-50%) were optimised. The procedure was repeated 15 times using different randomly selected subsets each time. The colour bar in each subplot shows the MCC mean scale, where values close to 1 represent more robust and consistent results. The MCC metric was positive in all cases.

**Supplementary Figure S3.** Sensitivity analysis for *SE*-Paw_max_ and *SE*-Flow_max_ features using a small grid search of *r* values around of 0.2 (0.15, 0.16, 0.17, 0.18, 0.19, 0.2, 0.21, 0.22, 0.23 0.24 and 0.25 x SD). Matthews correlation coefficient (MCC) from 15 repetitions upon optimization subset. The MCC index for *SE*-Flow_max_ feature did not change (0.84) for *r* values ranging between 0.15 to 0.24 but decrease for *r*=0.25 from 0.83 to 0.79. *SE*-Paw_max_ feature did not show substantial differences in MCC index (ranging between 0.84 to 0.86) at *r* values ranging between 0.15 to 0.2, whereas for *r*>0.21 the MCC index notably decreased until reaches an MCC=0.74. The sensitivity analysis suggested that *r*=0.2 represent a more robust local maximum for *SE*-Flow_max_ feature.

**Supplementary Table S1.** Performance of the algorithm in the 15 repetitions of the cross-validation procedure for *SE*-Flow_max_25 (*m*=2 and *r*=0.2) and *SE*-Paw_max_30 (*m*=4 and *r*=0.2). Median and interquartile range are represented.

|  | *SE*-Flow_max_25 | | *SE*-Paw_max_30 | |
| --- | --- | --- | --- | --- |
|  | Opt. | Val. | Opt. | Val. |
| MCC | 0.85 (0.82-0.85) | 0.78 (0.78-0.85) | 0.85 (0.85-0.88) | 0.85 (0.78-0.86) |
| Se | 0.91 (0.91-0.94) | 0.92 (0.86-0.93) | 0.94 (0.91-0.94) | 0.93 (0.93-1) |
| Sp | 0.91 (0.91-0.91) | 0.92 (0.92-0.93) | 0.94 (0.91-0.94) | 0.86 (0.84-0.92) |
| ACC | 0.92 (0.91-0.92) | 0.89 (0.89-0.93) | 0.92 (0.92-0.94) | 0.93 (0.89-0.93) |
| PPV | 0.91 (0.91-0.91) | 0.92 (0.92-0.93) | 0.94 (0.91-0.94) | 0.88 (0.87-0.93) |
| NPV | 0.91 (0.91-0.94) | 0.92 (0.86-0.93) | 0.94 (0.91-0.94) | 0.92 (0.92-1) |

**Supplementary Table S2.** Performance of the algorithm stratified by ventilator modality for *SE*-Flow_max_25 (*m*=2 and *r*=0.2) and *SE*-Paw_max_30 (*m*=4 and *r*=0.2).

|  | *SE*-Flow_max_25 | | *SE*-Paw_max_30 | |
| --- | --- | --- | --- | --- |
|  | PSV | ACV | PSV | ACV |
| MCC | 0.83 | 0.79 | 0.69 | 0.77 |
| Se | 0.95 | 0.83 | 0.82 | 0.75 |
| Sp | 0.87 | 0.96 | 0.87 | 0.99 |
| ACC | 0.91 | 0.89 | 0.84 | 0.87 |
| PPV | 0.88 | 0.95 | 0.86 | 0.99 |
| NPV | 0.95 | 0.85 | 0.83 | 0.79 |
